# Impacts of Aging Services: Insights from the National Surveys of AAAs and Title VI Programs

**DOI:** 10.1093/geroni/igaf122.3431

**Published:** 2025-12-31

**Authors:** Heather Menne, Krishnaa Nadig Nair, Kate Singer

**Affiliations:** Miami University, Oxford, Ohio, United States; Miami University, Oxford, Ohio, United States; Miami University, Oxford, Ohio, United States

## Abstract

Aging services operate within a shifting landscape shaped by the evolving needs and requirements of consumers and providers, legislative challenges, and external—often unpredictable—factors. The National Survey of Area Agencies on Aging (AAA) and the National Survey of Title VI Programs are conducted regularly to understand how AAAs and Tribal organizations are responding to the evolving needs of older adults and caregivers in communities around the country. This review identifies 26 publications that draw on data from the National Survey of Area Agencies on Aging (AAAs), with some also incorporating findings from the National Survey of Title VI programs. These publications cover six themes: COVID-19 (5 publications), healthcare and healthcare collaborations (6), general AAA operations (5), home and home modifications (3), communities (2), and staffing (1), and point to a system under strain, requiring more adaptable and equitable responses. Research on COVID-19 highlights heightened social isolation of older adults, disrupted healthcare access, and the critical role of AAAs in vaccine outreach and emergency support. Healthcare-related studies explore integrated care models, systemic inequities, and the power of community partnerships in expanding care access. These findings provide critical insights for policymakers, practitioners, and researchers, offering a foundation for informed decision-making and program development. Ensuring this data is used effectively can help strengthen aging services, making them more adaptable, inclusive, and better equipped to support older adults.

